# Enhanced Bone Regeneration by Schwann Cells through Coupling of Osteogenesis and Angiogenesis via β-catenin signaling in a Preclinical Model of Distraction Osteogenesis

**DOI:** 10.7150/ijms.100854

**Published:** 2025-01-01

**Authors:** Xiaoyu Wang, Yifan Yu, Rui Zhang, Jia Xu, Qinglin Kang

**Affiliations:** Department of Orthopaedic Surgery, Shanghai Sixth People's Hospital Affiliated to Shanghai Jiao Tong University School of Medicine, Shanghai, China.

**Keywords:** Distraction osteogenesis, Bone marrow mesenchymal stem cells, Schwann cells, Angiogenesis, Osteogenesis

## Abstract

**Background:** The lengthy period of external fixation for bone consolidation increases the risk of complications during distraction osteogenesis (DO). Both pro-angiogenic and osteogenic potential of bone marrow mesenchymal stem cells (BMSCs) contribute to bone regeneration during DO. The underlying mechanism of Schwann cells (SCs) in promoting bone regeneration during DO remains poorly understood.

**Methods:** The impacts of RSC-96 on the proliferation, migration, and osteogenic differentiation of BMSCs in the coculture system were investigated. The pro-angiogenic potential of BMSCs was evaluated by migration and tube formation assay. Quantitative real-time PCR was used to analyze angiogenic and osteogenic markers. ELISA was used to detect the secretion of various neurotrophins. Protein expressions of Activate protein kinase B (AKT)/β-catenin signaling were assessed by western blot. *In vivo*, dynamic expression levels of neurotrophic factors were detected in a preclinical rat DO model. Promotive effects of vascularization and mineralization provided by RSC-96 derived conditioned medium (CM) in a rat DO model were verified radiologically, biomechanically and histologically.

**Result:** Coculture system with RSC-96 promoted osteogenic ability of BMSCs, with increased cell viability, alkaline phosphatase staining, mineralized nodule formation, and osteogenic gene expression. Additionally, increased angiogenic gene expression of BMSCs and angiogenic capacity of endothelial cells demonstrated enhanced pro-angiogenic potential of BMSCs. Secretion of angiogenic and neurotrophic factors were enhanced in the coculture system. These effects were accompanied by activation of AKT/GSK-3β/β-catenin signaling, as evidenced by western blot analysis and the inhibitory effect of AKT inhibitor. The mRNA expression of neurotrophic factors peaked at the end of the distraction phase during DO. Furthermore, RSC-96 derived CM accelerated bone regeneration, resulting in improved biomechanical parameters, radiological features and histological manifestations, along with increased vascularization in the distraction area.

**Conclusion:** Through activation of AKT/GSK-3β/β-catenin signaling, SCs enhanced the coupled angio- and osteogenesis effects of BMSCs. The preclinical evidence demonstrates that SCs derived CM with increased neurotrophins secretion can be a promising treatment approach to accelerate bone regeneration in the DO process.

## Introduction

New bone formation within the osteotomy gap can be induced by gradually controlling the separation of osteotomized bone segments to the desired length during distraction osteogenesis (DO) 1, 2. The DO process comprises three sequential phases: the latency phase after osteotomy, the distraction phase for distracting the osteotomized bone segments, and the consolidation phase for bone mineralization 3, 4. Although the efficacy of DO in limb lengthening 2, 5, 6, deformity correction 7-9, and treatment of large bone defects 10-12 is widely recognized, the lengthy duration with external fixation in position for bone regeneration is prone to many problems, including joint stiffness and local infection 5, 8, 10. For this reason, it is essential to study the use of biological methods including the administration of stem cells, growth factors and bioactive agents for accelerating bone consolidation and reducing the external fixation period during the DO process 3, 13.

The peripheral nervous system participates in skeletal development and regeneration via a series of signaling pathways mediated through the secretion of neuropeptides, neurotrophins and growth factors, along with the contribution from neural resident cells 14, 15. Schwann cells (SCs), the glia surrounding myelinated nerves, are pivotal cells for peripheral nerve repair and regeneration 14, 16. Studies on mouse models have shown that digital tip regeneration and mandibular repair are both positively regulated by peripheral innervation dependent SCs through paracrine factors 17, 18. Previous studies have confirmed that SCs can express various factors such as Nerve Growth Factor (NGF), Vascular Endothelial Growth Factor (VEGF), neurotrophin-3 (NT-3) and Brain-Derived Neurotrophic Factor (BDNF) 16. Tissue engineering of innervated bone regeneration with various biomaterial designs for controlling growth factor secretion has emerged as promising approaches for orchestrating mineralization and innervation during bone repair 14, 19. However, the detailed mechanisms involved in the synergistic interaction of neural cells and bone cells in the physiological microenvironment remain unknown. Given the nerve regenerative potential of SCs with various paracrine factors involved and the close relationship between bone and nerve, SCs provide novel ideas for facilitating osteogenesis, thereby accelerating bone consolidation and shortening the external fixation period during DO treatment.

The abundant capillary system within bone tissue not only provides the network for oxygen and nutrients supply, but also ensures the paracrine effect of various growth factors and cytokines that are critical for tissue regenerative microenvironment 19. Previous studies have identified the mutually interdependent effects between angiogenesis and osteogenesis provided by bone marrow mesenchymal stem cells (BMSCs) within the vascularized osseous structure 13, 20. Several researches point to BMSCs' pro-angiogenic capacity and stimulatory effect of osteogenesis as promising treatment approaches for facilitating bone consolidation in the process of DO 1, 21. Although SCs show a range of cellular processes linked to nerve regenerative potential 16, 22, the impact and underlying mechanism of SCs on the pro-angiogenic and osteogenic potential of BMSCs remain poorly understood.

Studies have revealed the regulatory role of AKT (activate protein kinase B) signaling pathway in both osteogenic and angiogenic effects of various neurotrophic factors 23, 24. Phosphorylated AKT signal can activate downstream β-catenin that facilitates osteogenesis and suppresses osteoclastogenesis 21, 25, 26. Furthermore, study has found that exogenous application of NGF in mice significantly increases limb bone formation under mechanical stress, along with enhanced β-catenin activity in osteoblasts 27. Xue et al. used 7,8-Dihydroxyflavone as TrkB agonist to mimic the functions of BDNF. They demonstrated that BDNF could enhance bone formation via the activated Wnt/β-catenin pathway 28. In addition, enhanced AKT phosphorylation level was observed in BDNF-induced osteoblasts migration for fracture healing 24. As NGF and BDNF can be secreted by SCs 15, 16, we hypothesized that the coupled angio- and osteogenic effects in BMSCs may be strengthened by SCs through AKT/β-catenin signaling axis.

The purpose of this research was to explore the impacts of SCs on BMSCs' pro-angiogenic potential and osteogenic capacity and the role of AKT/β-catenin in this process. A preclinical rat DO model was used to investigate the dynamic expression level of SC-related neurotrophins for determining the appropriate time point for local administration. The curative prospect of SCs derived bioactive substances in accelerating consolidation by facilitating neovascularization and mineralization was then studied in the rat DO model.

## Materials and Methods

### Culture of cells

Human umbilical vein endothelial cells (HUVECs) were cultured in Dulbecco's modified Eagle's medium (DMEM; Gibco, US) supplemented with 10% (v/v) fetal bovine serum (FBS; Gibco, US) and 1% (v/v) penicillin-streptomycin (P/S; Gibco, US). Femurs and tibias obtained from two-week-old male Sprague-Dawley (SD) rats were collected to harvest BMSCs through bone marrow flushing. Modified Eagle's medium α (α-MEM; Gibco, US) containing P/S [at a concentration of 1% (v/v)] and FBS [at a concentration of 10% (v/v)] was used for culturing the isolated BMSCs. The cultured cells underwent trypsin (Gibco, US) treatment and were replated for passage after reaching the cell confluence of 80% to 90%. Further experiments were performed with the BMSCs at the 2^nd^ to 4^th^ passage. Complete DMEM containing 1% (v/v) P/S and 10% (v/v) FBS was used for culturing Schwann cell line RSC-96 (American Type Culture Collection, US). Further experiments were performed with the cells at the logarithmic growth phase. Coculture system of BMSCs and RSC-96 (BMSCs-RSC-96) was constructed using transwell plates (Corning Incorporated, US) with 0.40 µm sized pores. RSC-96 and BMSCs were seeded onto the upper and lower chamber, respectively. Cell culture was conducted at humidified environment under 37°C with 5% CO_2_. Additionally, 100 nM MK2206 (MedChemExpress, US) was used as AKT signaling inhibitor in further experiments for confirming the mechanism through which RSC-96 cells regulate the function of BMSCs and HUVECs.

### Cell counting kit-8 (CCK-8) and 5-ethynyl-2′-deoxyuridine (EdU) labeling

The lower chambers of the Transwell plates (96 wells) were seeded with BMSCs (2×10^3^ in each well). The BMSCs were treated with serial relative amounts of RSC-96 cells at the upper chambers (the ratios of the number of RSC-96 cells to BMSCs were 0, 0.5, 1, 2, 3 and 4 :1, respectively). The cells underwent 24, 48, 72 or 96 hours of incubation before assessing the cell viability. For performing CCK-8 assay, BMSCs were treated for additional 120 minutes with CCK-8 reagents (10 μL; Dojindo, Japan) and freshly prepared medium (90 μL). Then, a micro-plate reader (ThermoFisher, US) was used for measuring absorbance at 450 nm.

5-Ethynyl-2'-deoxyuridine (EdU) labeling assay was carried out using the BeyoClick™ EdU Cell Proliferation Kit with Alexa Fluor 555 (Beyotime, China). The cells were incubated with EdU solution for 2 hours, fixed with 4% PFA for 15 minutes and permeabilized with 0.3% Triton X-100 for 15 minutes. After washing with PBS, cell incubation using the click reaction mixture was carried out in the dark for 30 minutes. Finally, the nuclei were counterstained with DAPI. The percentage of EdU-positive cells was analyzed using a fluorescence microscope (Leica DMi8, Germany).

### Osteogenic differentiation

Osteogenic differentiation was assessed by the staining of alizarin red S (ARS) and alkaline phosphatase (ALP). Briefly, BMSCs (3×10^4^/well) were seeded in 24-well plates. After reaching 80% confluence, cell incubation was conducted under osteogenic induction medium (OIM; Cyagen, China) in each treatment group. The impact of SCs on BMSCs' osteogenic capacity was investigated through coculture of RSC-96 and BMSCs during osteogenic differentiation. The underlying mechanism was verified using 100 nM MK2206. ALP staining was carried out using dye solution (Beyotime, China) seven days after osteogenic induction. An optical microscopic device (Nikon TE2000-E, Japan) was used for viewing the stained image. The image processing software ImageJ (National Institutes of Health, US) was used to measure the positive staining area. For quantitatively evaluating the mineral deposition, ARS staining (Cyagen, China) was conducted 14 days after osteogenic induction and the absorbance at OD 570 nm was measured after calcium elution using cetylpyridinium chloride at a concentration of 10% (w/v) (Sigma-Aldrich, US).

### Preparation of conditioned medium (CM)

BMSCs were treated under completed modified Eagle's medium alpha, osteogenic induction medium, or osteogenic induction medium with coculture of RSC-96 (RSC-96 : BMSCs = 2 : 1) according to different grouping conditions. The medium was changed to complete medium on the 7^th^ day during osteogenic differentiation and the cells were incubated for another 48 hours. AKT specific inhibitor MK2206 (100 nM) was added to the complete medium for confirming the underlying mechanism through which RSC-96 cells regulate the function of BMSCs and HUVECs in further study. Following the harvesting of CM from each group (CM-α-MEM for the BMSCs incubated in α-MEM complete medium; CM-OIM for the BMSCs incubated in osteogenic induction medium; CM-RSC-96 for the BMSCs cocultured with RSC-96; CM-MK2206 for the BMSCs cocultured with RSC-96 and MK2206), the supernatant was collected by centrifuging at 2×10^3^ g for 10 minutes and then kept in -80℃ for subsequent investigations.

### Cell migration assay

HUVECs were tested for determining the migration ability using Transwell and scratch wound assay. HUVECs seeded in the 6-well plate were cultivated till reaching the confluence. Then the tip of the sterilized pipette was used for scratching the confluent cell layer. After that, the cells were washed and treated under varying conditions of CM in serum-free medium. MK2206 (100 nM) was used as AKT signaling inhibitor for confirming the mechanism through which RSC-96 cells influence the cell migration ability of HUVECs. Microscopic photographs of wound areas were obtained immediately, 6 and 12 hours after the assay. The photographs were analyzed by ImageJ for calculating migration area. Transwell plates containing 24 wells were used for Transwell migration assay of HUVECs. A total of 2 × 10^4^ cells in the upper chamber with the pores sized 8 µm were cultured with 200 μL serum-free DMEM. The lower chamber was filled with 500 μL CM from different groups. The cells remaining at the upper side of the membrane were swabbed away after 12 hours. 4% (w/v) paraformaldehyde (Solarbio, China) was used for fixation of the migrated cells. After that, the cells underwent 10 minutes' staining with crystal violet (Solarbio, China; 0.5% (w/v)) before being photographed with an optical microscope (Nikon TE2000-E, Japan). ImageJ software was used for counting the migrated cells.

### Tube formation assay

To evaluate BMSCs' pro-angiogenic capacity, HUVECs were pretreated with CM obtained from different groups for six hours and then incubated in 96-well plates (2 × 10^4^ /well) precoated with Matrigel (Corning, US) for an additional 6 hours. An optical microscope (Nikon TE2000-E, Japan) was used for viewing the capillary network. The software ImageJ was used to measure the number of branch points and tube length.

### Enzyme-linked immunosorbent assay (ELISA)

VEGF, NGF, BDNF and NT-3 concentrations in the CM from each group were analyzed using commercial ELISA kits (Abcam, US, ab100786 for VEGF; Boster, US, EK0471 for NGF; Abcam, ab213899 for BDNF; Boster, EK0474 for NT-3). The experiment was performed under the manufacturer's instruction. The concentration was determined by the standard curves.

### Extraction of cellular RNA and quantitative real-time PCR analysis

On the 7^th^ day after osteogenic induction of BMSCs, total cellular RNA extraction was carried out using RNA Purification Kit (EZBioscience, US). 500 ng purified RNA was then utilized to synthesize cDNA with Reverse Transcription Kit (EZBioscience, US). After that, SYBR Green Master Mix (EZBioscience, US) was used for quantitative Real-Time PCR (qRT-PCR). Glyceraldehyde-3-phosphate dehydrogenase (GAPDH) was used as a reference for normalization. The relevant sequences of the primers (BioTNT, China) are shown in Table 1. 2^-ΔΔCT^ method was used to determine the relative expression of genes.

### Western blot analysis

7 days after osteogenic induction of BMSC, RIPA lysis buffer (Solarbio, China) along with the inhibitors against protein phosphatase and protease was applied for obtaining the total protein. BCA kit (EpiZyme, China) was utilized for measuring the quantity of total protein. Seperation of the protein samples (30µg/group) were performed with 10% (w/v) SDS-PAGE. After transferring the protein to the polyvinylidene difluoride membranes (Millipore, US), the protein-attached membranes were blocked by 5% (w/v) bovine serum albumin for 60 minutes under normal temperature. After that, the primary antibodies against AKT, phosphorylated AKT (p-AKT; Ser473), glycogen synthase kinase-3β (GSK-3β), phosphorylated GSK-3β (p-GSK-3β; Ser9), β-catenin, phosphorylated β-catenin (p-β-catenin; Ser675), and GAPDH were used for incubation at 4°C overnight. A secondary antibody (1:2000, ab6721, Abcam) conjugated to horseradish peroxidase (HRP) was then used for membrane incubation under room temperature. 60 minutes later, enhanced chemiluminescence solution (Millipore, US) was applied for visualizing the bands on the membranes. Grayscale evaluation with ImageJ (National Institutes of Health, US) was used for semiquantitative analysis of the bands (n = 3).

### Animal model and experiments

All experimental procedures were approved by the Animal Welfare Ethics Committee of Shanghai Sixth People's Hospital Affiliated to Shanghai Jiao Tong University School of Medicine and were in line with the guidelines of the Animal Research: Reporting of *In vivo* Experiments (ARRIVE). We have adhered to the ARRIVE guidelines and have supplied the checklist. In this study, 42 adult SD rats (male) weighing between 350 and 400g were used and subjected to two sections of animal study at random. 18 rats were used in the first section to determine the local expression levels of SCs relevant neurotrophic factors during various stages of DO. 24 rats were included in the second section to assess the therapeutic outcomes of CM derived from coculture system of RSC-96 and BMSCs in bone regeneration during DO. The sample size was determined according to the Resource Equation Approach 29. Under normal temperature (22 ± 2°C), the rats were fed in cages with a twelve-hour cycle of light and darkness. Pelleted diet and drinking water were available at all times during their housing in cages.

In the first section of animal study, following surgical incision under general anesthesia, transverse osteotomies with the periosteum preserved as much as possible, were made at the middle of right tibiae. After that, the osteotomized tibial segments were fixed by a custom-designed monorail external fixation device (Xinzhong Company, China) before layerwise suture of operative incisions. DO treatment composes of three sequential phases: latency phase for 5 days, distraction phase for 10 days (0.25 mm every 12 hours), and consolidation phase for 28 days. At day 0, 5, 10, 15, 29, and 43 after osteotomy, the rats (n = 3 on each day) were sacrificed for obtaining tibia samples.

In the second section of animal study, twenty-four rats were subjected to the SCs-CM (n = 12) and control (n = 12) groups at random and received the same surgical and DO treatment procedure as described in the first section. 100μL CM derived from coculture system of RSC-96 and BMSCs (BMSCs-RSC-96; RSC-96 : BMSCs = 2 : 1) or an equal volume of DMEM complete medium were percutaneously injected at the distraction gap every 3 days under anesthesia at consolidation phase, respectively. At the 14^th^ and the 28^th^ day of the consolidation phase, the rats underwent DO treatment received euthanasia through injection of 2% pentobarbital with excessive dose of 200 mg/kg. Bilateral tibias were then harvested at each time point (n = 6 in each group) for further experiments.

### Tissue RNA extraction and qRT-PCR analysis

The regenerate callus with intact periosteum in the distraction gap, along with 3 mm of the proximal and distal bone segments were obtained. After being weighed, the specimens were kept in liquid nitrogen for subsequent experiments. Total RNA of each specimen was then extracted with TRIzol reagent kit (Invitrogen, US). Reverse Transcription Reagent Kit (EZBioscience, US) was used for cDNA synthesis. After that, SYBR Green Master Mix (EZBioscience, US) was used for qRT-PCR according to standard protocols. Relevant sequences of the primers (BioTNT, China) are shown in Table 1. 2^-ΔΔCT^ method was used to determine the relative expression of genes. Target gene expressions were normalized according to the expression of GAPDH.

### X-ray radiograph and micro-computed tomography (micro-CT)

From the start of the consolidation phase, X-ray radiographs of the distraction gap were taken once a week with the voltage of 32 kV and exposure time of 6000 ms. 2 and 4 weeks after the beginning of consolidation phase, the tibias were harvested for micro-CT scanning (n = 5 per group at each time point). The micro-CT device (Skyscan 1076, Bruker, Belgium) was operated with 80 kV voltage, 112 μA current, 370 ms exposure time, and 18 μm thickness per slide. For quantitative evaluation of regenerated callus, the software CTVox (Bruker) was used for reconstructing the obtained tibias in 3 dimensions. Measurements of bone volume/tissue volume (BV/TV) and bone mineral density (BMD) were conducted with CTAn software (Bruker).

### Biomechanical testing

During biomechanical testing, the samples (harvested at the 28^th^ day of consolidation phase, n = 5 per group) were loaded anteriorly-posteriorly under 250N loading cells on the 4-point bending equipment (Hounsfield Test Equipment, UK). The long axis of the tibias was aligned perpendicular to the blades. The load with a displacement rate of 5 mm/min was applied constantly to the distraction zone. Using Vernier Graphical Analysis software (Vernier, US), biomechanical properties of the regenerate callus including ultimate load, energy to failure, and modulus of elasticity (E-modulus) were measured and were normalized according to the properties of the contralateral tibias (presented as %).

### Histological and immunohistochemical analyses

Immediately after micro-CT analysis, the tibia specimens harvested at the 14^th^ and the 28^th^ day of the consolidation phase underwent fixation with 4% (w/v) PFA for 24 hours. Decalcification of the specimens were conducted in 10% (w/v) ethylenediaminetetraacetic acid for the next 3 weeks. The decalcified specimens were dehydrated by series concentration of ethanol, followed by paraffin embedding. Each specimen was sliced lengthwise into the slices with 5µm thickness for Safranine O-Fast Green (SO-FG), Masson's trichrome, and Hematoxylin and eosin (H&E) staining (n = 3 in each group at each time point).

Local expression of protein was measured by immunohistochemistry (n = 3 in each group). Activities of endogenous peroxidase within the sections were quenched by 0.3% (v/v) hydrogen peroxide. After that, the sections underwent retrieval of antigen for 20 minutes at 95℃ in citrate buffer (0.01 mol/L, pH 6.0). and were blocked with 5% (v/v) goat serum for 60 minutes. Primary antibodies included anti-β-catenin (1:200; no.8480, Cell Signaling Technology), anti-osteocalcin (OCN) (1:200; A6205, ABclonal, China), and anti-VEGF (1:150, A0280, ABclonal) antibodies were then used for incubation at 4℃ overnight. Thereafter, secondary antibody (1:1000, ab6721, Abcam) conjugated to HRP was used for the subsequent 60 minutes' incubation under normal temperature before counterstaining by hematoxylin. At last, the HRP-streptavidin system (Dako, Denmark) was utilized for visualization and detection.

### Statistical analysis

GraphPad Prism 8 software (GraphPad Software, US) was used for statistical analysis. The data were presented as mean ± standard deviation. Statistical analysis between two groups was determined by Student's t-test. One-way analysis of variance (ANOVA) followed by Tukey's post hoc test was used for comparing the data from three or more groups. Two-way ANOVA was used for statistical comparison across three or more groups over time. A two-tailed *P*-value of <0.05 was regarded as statistically significant.

## Results

### RSC-96 promoted the prolifration, osteogenic differentiation and pro-angiogenic potential of BMSCs

**Figure 1:**
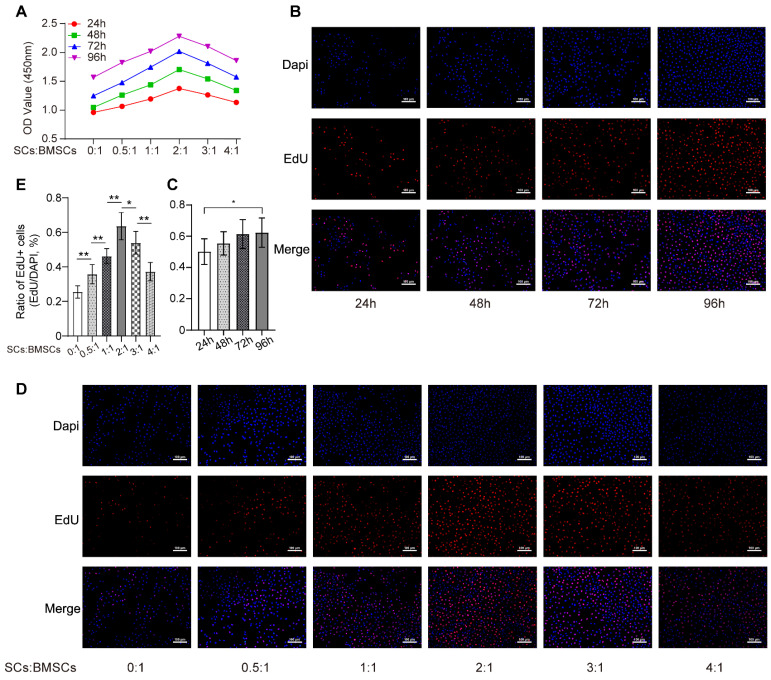
Effects of RSC-96 on proliferation ability of BMSCs. **(A)** Proliferation of BMSCs incubated with different number of RSC-96 after 24, 48, 72 and 96 hours was analyzed using CCK-8 assay. Two-way ANOVA with Tukey's multiple comparisons test was used. **(B-E)** Cell proliferation analysis of BMSCs measured by EdU labeling assay. Scale bar: 100 µm. Representative images **(B)** and quantitative percentages **(C)** of EdU positive BMSCs incubated with RSC-96 (the ratio of the number of RSC-96 to BMSCs was 2 :1) after 24, 48, 72 and 96 hours. Representative images **(D)** and quantitative percentages **(E)** of EdU positive BMSCs incubated with serial relative amounts of RSC-96 cells after 96 hours. One-way ANOVA with Tukey's multiple comparisons test was used. The results are presented as mean ± standard deviation. *P < 0.05; **P < 0.01.

**Figure 2:**
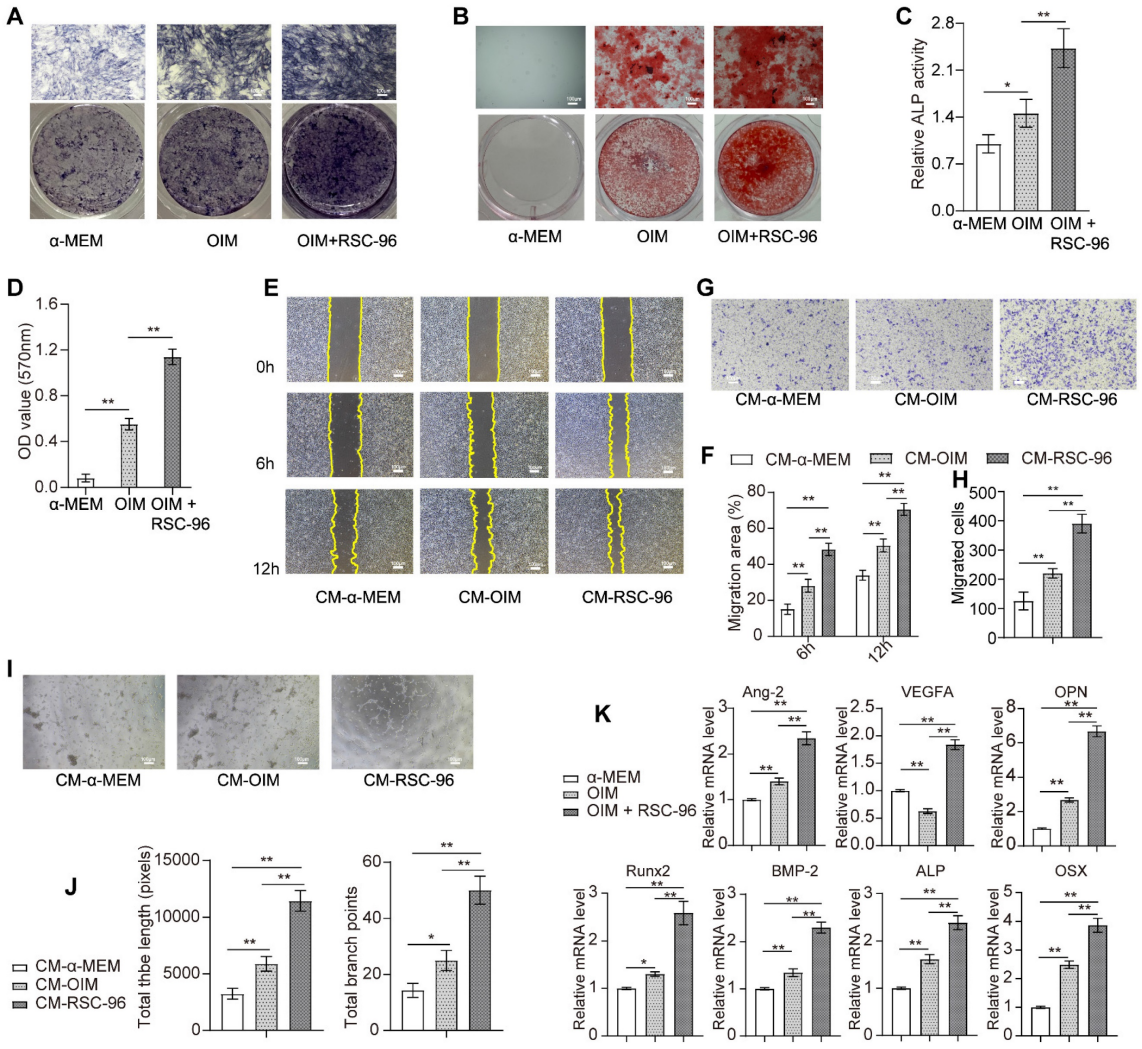
Effects of RSC-96 on BMSCs' pro-angiogenic capacity and osteogenesis. **(A-D)** Osteogenic activity of BMSCs treated with vehicle, OIM and RSC-96 was detected via ALP staining **(A)** after 7 days and ARS staining **(B)** after 14 days. Quantitative analysis of ALP staining by ALP activity assays **(C)**. Quantitative analysis of calcium deposition through measurement of optical density **(D)**. Scale bar: 100 µm. **(E-H)** Scratch wound assay **(E, F)** and Transwell migration assay **(G, H)** for detecting the migration capacity of HUVECs stimulated with CM from BMSCs treated with vehicle, OIM and OIM + RSC-96. Scale bar: 100 µm. **(I, J)** Typical photos **(I)** and quantification **(J)** of tube-shaped structure in HUVECs from different groups. Scale bar: 100 µm. **(K)** Osteogenic- and angiogenic-specific gene expression in BMSCs from each group. One-way analysis of variance (ANOVA) followed by Tukey's post hoc test was used. The results are presented as mean ± standard deviation. *P < 0.05; **P < 0.01.

As shown in Figure 1A, an increased number of RSC-96 had significant stimulatory effects on BMSCs proliferation and reached optimal cell viability when the ratio of the number of RSC-96 to BMSCs was 2 :1. This effect was mitigated by increasing the RSC-96 number even further. Similarly, the percentage of BMSCs labeled with EdU moderately increased after 24, 48, 72 and 96 hours, when the ratio of the number of RSC-96 to BMSCs was 2 :1 (Figure 1B, C). The proportion of EdU-positive BMSCs incubated with RSC-96 significantly increased and reached the highest when the ratio of the number of RSC-96 to BMSCs was 2 :1 (Figure 1D, E). Therefore, a 2 :1 ratio of the number of RSC-96 cells to BMSCs was employed in further experiments. ARS and ALP staining were used to assess osteogenic ability of BMSCs at the 7^th^ and 14^th^ day during osteogenic differentiation (Figure 2A-D). Enhanced ALP activity of BMSCs was observed when the ratio of the number of RSC-96 to BMSCs was at 2 :1 in the coculture system (Figure 2A, C). ARS staining (Figure 2B) and quantitative evaluation of mineralized nodules (Figure 2D) demonstrated that RSC-96 coculture could induce enhanced mineralization. To investigate the effect of RSC-96 on pro-angiogenic potential of BMSCs, CM in different groups was used to treat HUVECs for angiogenesis-related experiments. Scratch assay (Figure 2E, F) and transwell assay (Figure 2G, H) indicated that migration capacity of HUVECs was improved by CM-OIM and was further promoted by CM-RSC-96. HUVECs stimulated by CM-OIM exhibited an increased quantity of structures resembling capillary tubes in comparison to those stimulated by CM-α-MEM (Figure 2I), with an increased number of branch points and total tube length (Figure 2J). CM-RSC-96 treatment resulted in the formation of optimal capillary tube-like structures (Figure 2I, J), indicating a synergistic promotive effect of OIM and RSC-96 on the pro-angiogenic potential of BMSCs. QRT-PCR analysis showed that gene expression of osteogenic markers (*Runx2*, *BMP-2*, *OSX*, *OPN* and *ALP*) in BMSCs was promoted by OIM and further increased by additional RSC-96 treatment. Expression levels of angiogenesis-specific genes in BMSCs were assessed after osteogenic induction and RSC-96 treatment. QRT-PCR results confirmed that RSC-96 further promoted *Ang-2* and *VEGF* expressions in BMSCs (Figure 2K).

### RSC-96 enhanced coupled effect of angiogenesis and osteogenesis and production of neurotrophic factors via AKT/β-catenin pathway

**Figure 3:**
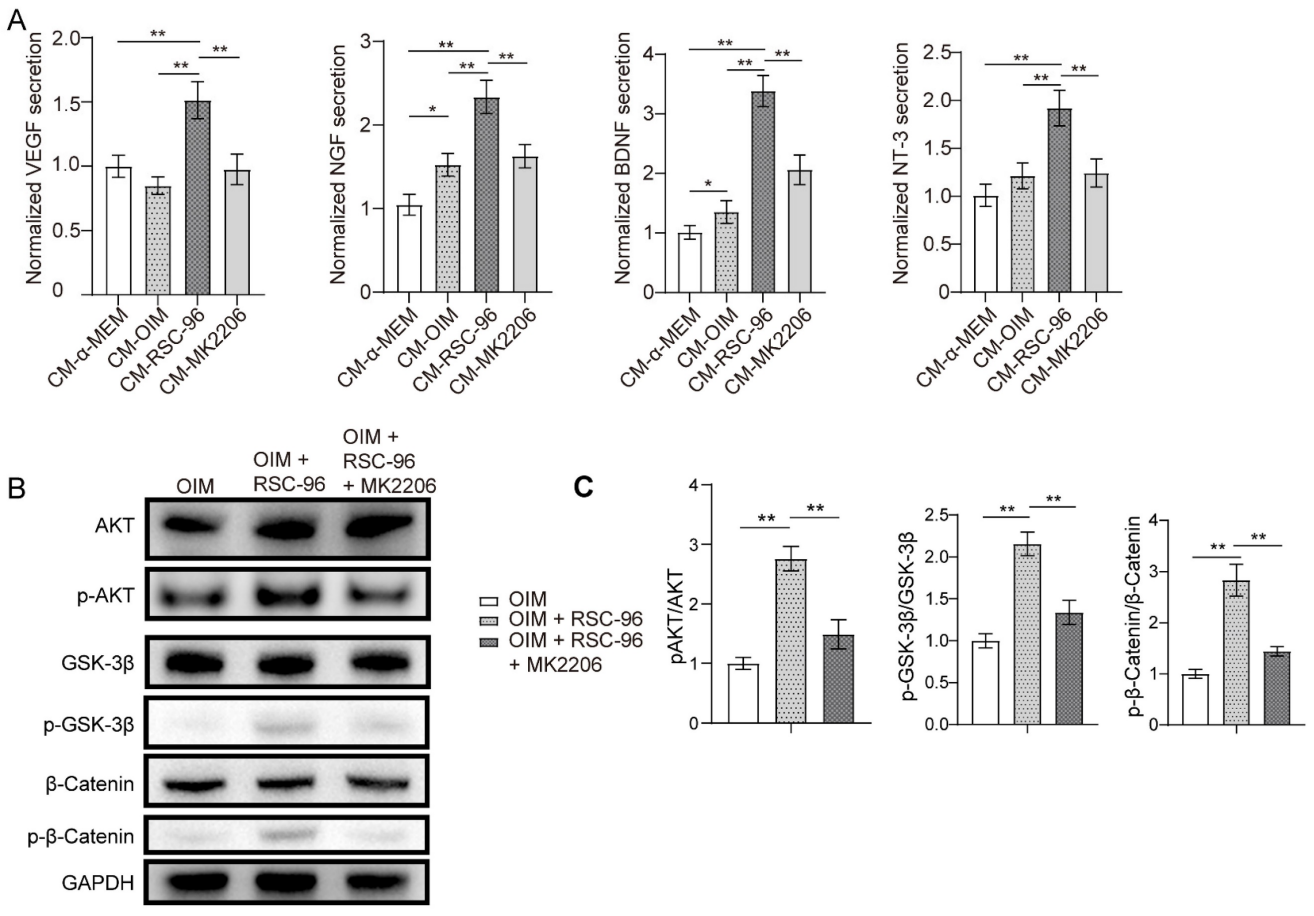
RSC-96 enhances the production of neurotrophic factors and activates the AKT/GSK-3β/β-catenin pathway in BMSCs. **(A)** ELISA measurement of NGF, BDNF and NT-3 concentrations in CM from BMSCs treated with vehicle, OIM, OIM + RSC-96 and OIM + RSC-96 + MK2206. **(B)** Protein expression level of AKT, p-AKT (Ser473), GSK-3β, p-GSK-3β (Ser9), β-catenin, and p-β-catenin (Ser675) in BMSCs treated with OIM, OIM + RSC-96 and OIM + RSC-96 + MK2206. The uncropped blots are presented in supplementary Figure S1. **(C)** Quantification of the phosphorylation level of AKT, GSK-3β, and β-catenin relative to GAPDH. The results were provided as the mean ± standard deviation and were evaluated by one-way analysis of variance (ANOVA) followed by Tukey's post hoc test. *P < 0.05; **P < 0.01.

ELISA was used to examine the production of neurotrophic and angiogenic factors in the CM from each group (Figure 3A). The results demonstrated that VEGF, NGF, NT-3 and BDNF secretions increased markedly in CM-OIM group and were further enhanced in CM-RSC-96 group, when compared to CM-α-MEM group. Moreover, repressed secretions of VEGF, NGF, BDNF and NT-3 through AKT inhibitor MK2206 was observed via ELISA, which indicated AKT pathway's contribution to these effects.

Western blot suggested that coculture with RSC-96 activated AKT and downstream signaling molecule GSK-3β/β-catenin in OIM induced BMSCs, based on the elevated phosphorylation levels at Ser473 of AKT, Ser9 of GSK-3β, and Ser675 of β-catenin (Figure 3B, C). AKT-specific inhibitor MK2206 significantly attenuated the phosphorylation status of GSK-3β/β-catenin. As shown by the ELISA results in Figure 3A, AKT/β-catenin signaling contributed to enhanced production of neurotrophic factors under the coculture system of BMSCs and RSC-96.

**Figure 4:**
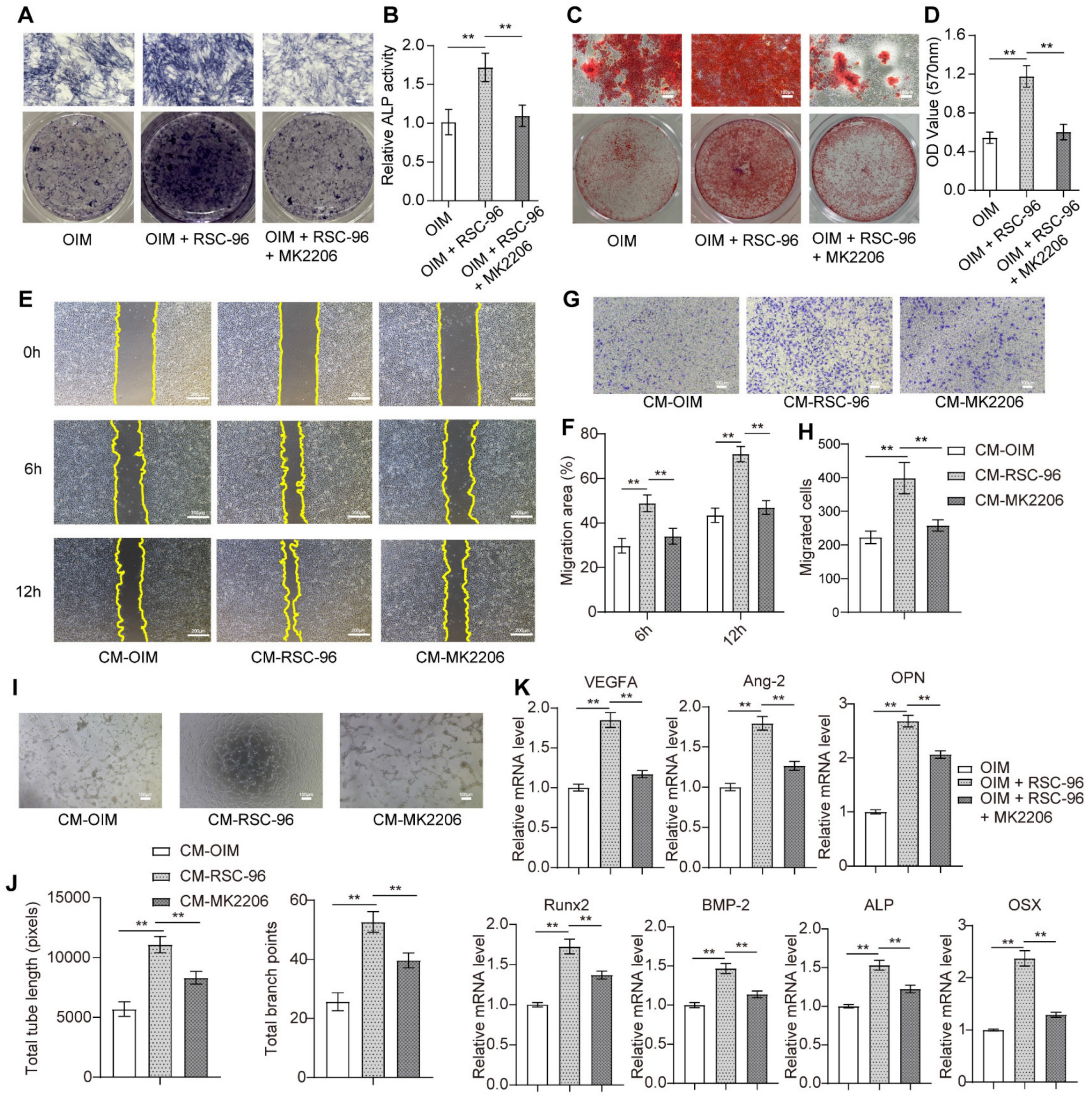
Inhibitory effects of MK2206 on RSC-96 induced pro-angiogenic potential and osteogenic differentiation of BMSCs. **(A, B)** Osteogenesis of BMSCs treated with OIM, OIM + RSC-96, and OIM + RSC-96 + MK2206 for 7 days was detected via ALP staining **(A)** and quantitative activity analysis **(B)**. Scale bar: 100 µm. **C, D** Calcium deposition in BMSCs after 14 days of treatment was evaluated by ARS staining **(C)** and measurement of optical density **(D)**. Scale bar: 100 µm. **(E, F)** Scratch wound assay for detecting the migration capacity of HUVECs stimulated with the CM from BMSCs treated with OIM, OIM + RSC-96, and OIM + RSC-96 + MK2206. Scale bar: 200 µm. **(G, H)** Transwell migration assay for detecting the migration capacity of HUVECs stimulated with the CM from BMSCs in different groups. Scale bar: 100 µm. **(I, J)** Representative images **(I)** and quantitative evaluation **(J)** of tube-like structures in HUVECs from different groups. Scale bar: 100 µm. **(K)** Angiogenic- and osteogenic-specific gene expression in BMSCs from different groups. The results were provided as the mean ± standard deviation and were evaluated by one-way analysis of variance (ANOVA) followed by Tukey's post hoc test. *P < 0.05; **P < 0.01.

AKT-specific inhibitor MK2206 was used to confirm the inhibitory impact of attenuated AKT signaling on pro-angiogenic capacity and osteogenesis in BMSCs when treated with RSC-96 (Figure 4). As expected, ALP (Figure 4A, B) and ARS (Figure 4C, D) assays showed repressed ALP activity and decreased calcium nodules after cocultured with RSC-96 and MK2206 compared to cocultured with RSC-96 alone. The results of the wound scratch assay (Figure 4E, F) and Transwell assay (Figure 4G, H) revealed repressed migration area and migrated cell number, indicating impaired cell migration capacity after CM-MK2206 treatment. Moreover, CM-MK2206 attenuated the CM-RSC-96-induced increase in the number of capillary tube-like structures of HUVECs (Figure 4I, J). QRT-PCR demonstrated similar inhibitory impacts on angiogenesis and osteogenesis related gene expression. (Figure 4K). These findings indicate that RSC-96 promotes the coupling of osteogenic and angiogenic process in BMSCs via activating AKT/GSK-3β/β-catenin signaling.

### Dynamic expression changes of multiple neurotrophic factors during DO

**Figure 5:**
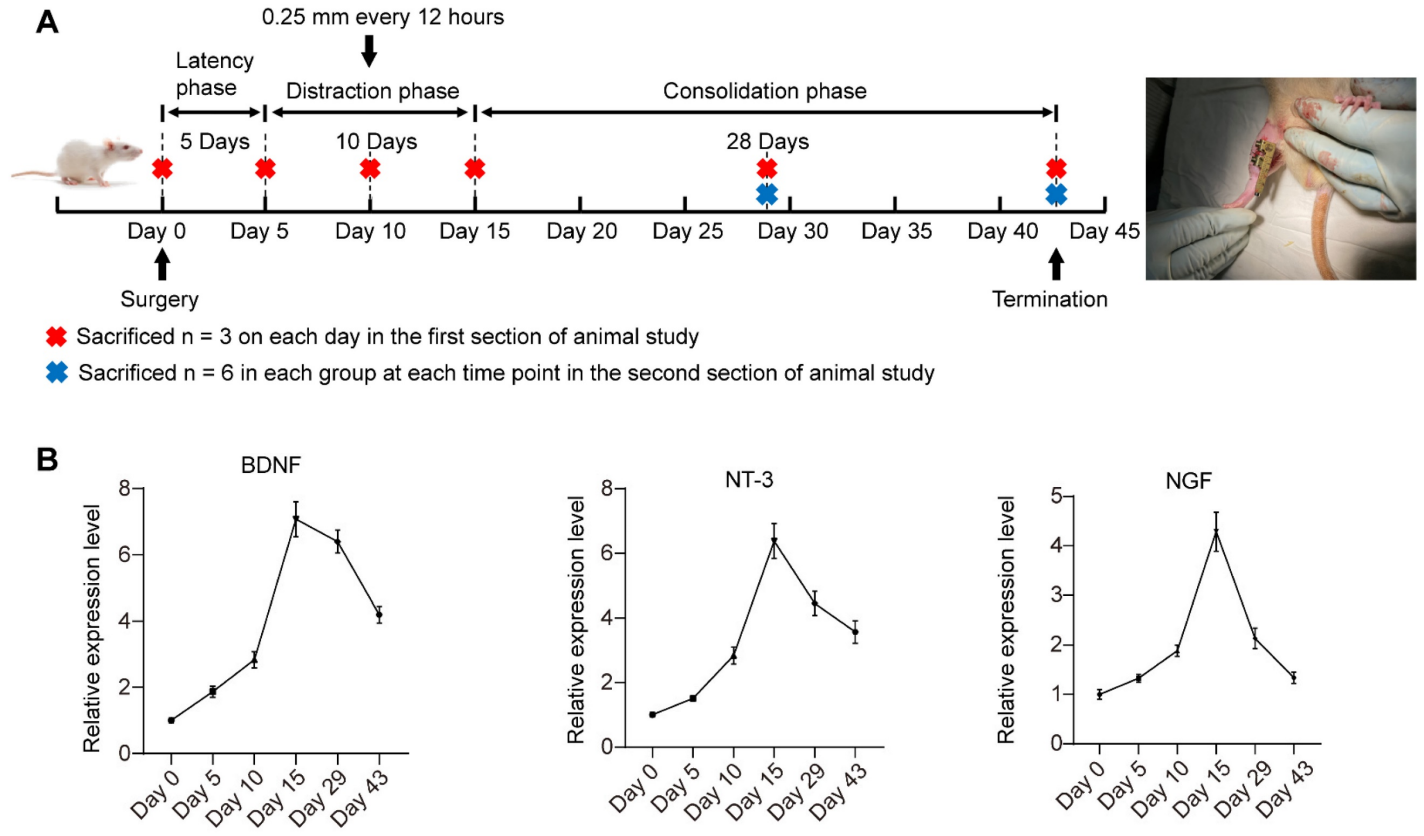
Timeline of DO treatment and dynamic changes in the expression of multiple neurotrophic factors during DO. **(A)** DO treatment that composed of three sequential phases: latency phase (5 days), distraction phase (10 days, with the lengthening rate of 0.25 mm every 12 hours), and consolidation phase (28 days). **(B)** The dynamic expression of NGF, NT-3 and BDNF in the tibial specimens during DO were detected by qRT-PCR. The tibial callus was harvested at different observation time points. One-way ANOVA with Tukey's post hoc test was used. All the data are presented as the mean ± standard deviation. *P < 0.05; **P < 0.01.

Figure 5A shows the timeline of animal experiments. The mRNA expression levels of multiple neurotrophic factors (NGF, NT-3 and BDNF) in the regenerate callus obtained at various observation time points were detected via qRT-PCR (Figure 5B). BDNF, NT-3 and NGF expression gradually increased at latency phase. Thereafter, BDNF, NT-3 and NGF expression significantly increased to different degrees during distraction, peaking at the end of the distraction phase (day 15). During the initial stage of consolidation phase, the increase in BDNF expression level remained greater than that of NT-3 and NGF. On day 43, the end of the consolidation phase, NGF expression returned to the level close to the baseline level at day 0. NT-3 and BDNF expression level remained high after 28 days of consolidation phase. On the basis of the lowered expression level of SCs-related neurotrophins detected at the consolidation phase, local application of SCs-CM at the beginning of the DO consolidation phase may ensure the effective level of these paracrine factors for facilitating bone regeneration.

### BMSCs-RSC-96 derived CM accelerated bone consolidation during distraction osteogenesis

**Figure 6:**
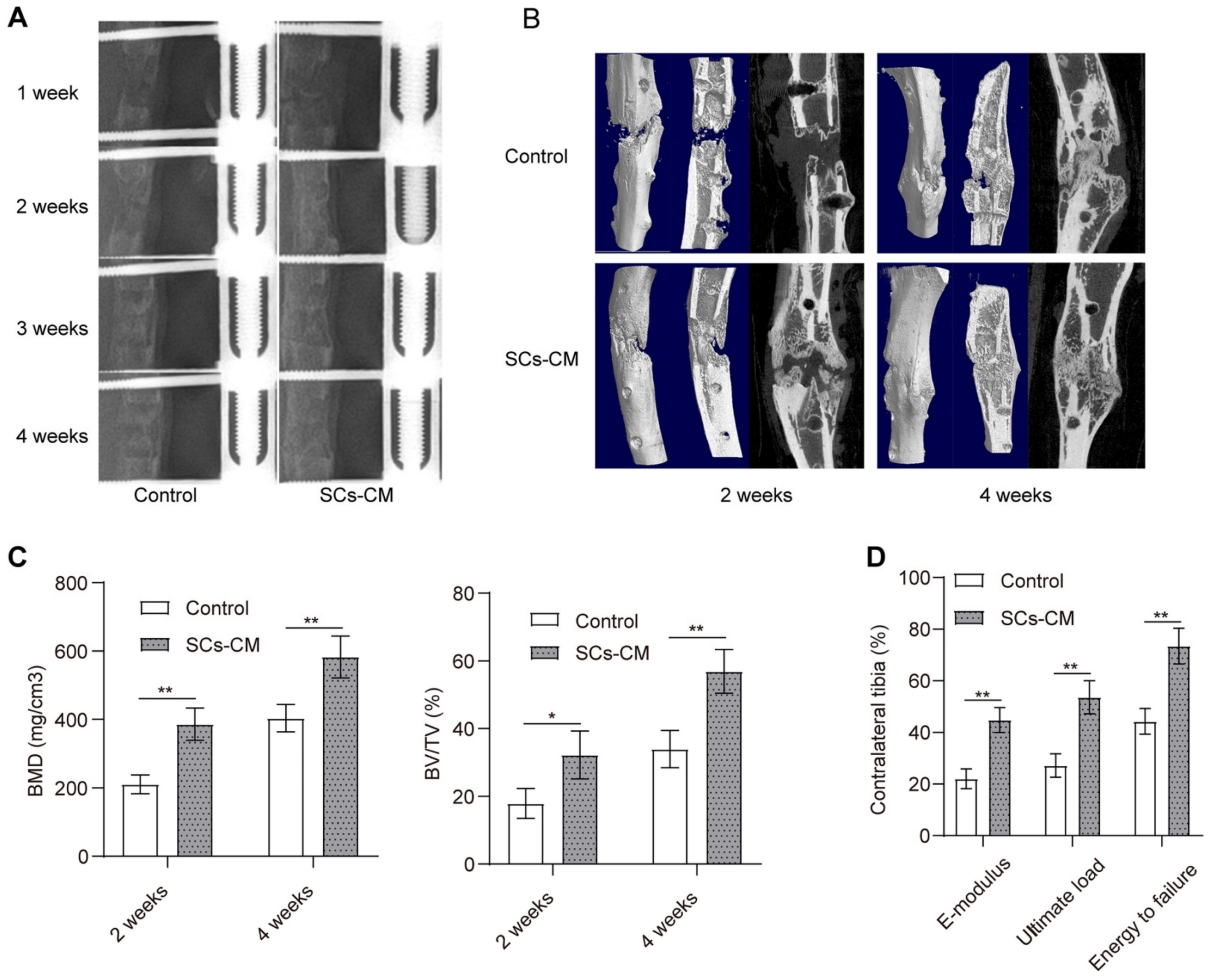
Accelerated bone consolidation via BMSCs-RSC-96 derived CM administration in a rat DO model. **(A)** Representative X-ray images showing dynamic changes of bone consolidation at various time points. **(B)** Three-dimensional reconstruction of the micro-CT data obtained at the 2^nd^ and the 4^th^ week of the consolidation phase. **(C)** Quantitative analysis of the micro-CT data including bone volume/tissue volume and bone mineral density. **(D)** Mechanical parameters, such as E-modulus, energy to failure, and ultimate load, for determining the quality of the newly mineralized regenerate in control and SCs-CM groups. The measurements were normalized (presented as %) according to the properties of the contralateral tibias. The results between the two groups were provided as mean ± standard deviation and evaluated by Student's t-test. *P < 0.05; **P < 0.01.

Progression of bone regeneration was shown by the X-ray radiographs (Figure 6A) obtained during consolidation phase. Both groups showed little sign of callus formation in the distraction area at the end of distraction phase. After that, bone consolidation appeared overtime in both groups with more mature calluses observed in the SCs-CM group with favorable continuity and volume. The difference was significant at the end of the consolidation phase. Micro-CT scanning manifested the similar observations of the distraction zone in two groups (Figure 6B). BV/TV and BMD of the regenerate callus obtained from SCs-CM group was markedly higher compared to that from control group at the 2^nd^ and 4^th^ week of the consolidation phase, indicating the contributive impact of bone regeneration provided by BMSCs-RSC-96 derived CM in the rat DO model (Figure 6C). Moreover, improved biomechanical properties of tibia specimens in SCs-CM group, including improved energy to failure, ultimate load, and E-modulus (Figure 6D), indicated the regenerative effect of BMSCs-RSC-96 derived CM during DO.

### Vascularized bone regeneration enhanced by BMSCs-RSC-96 derived CM within the distraction area

**Figure 7:**
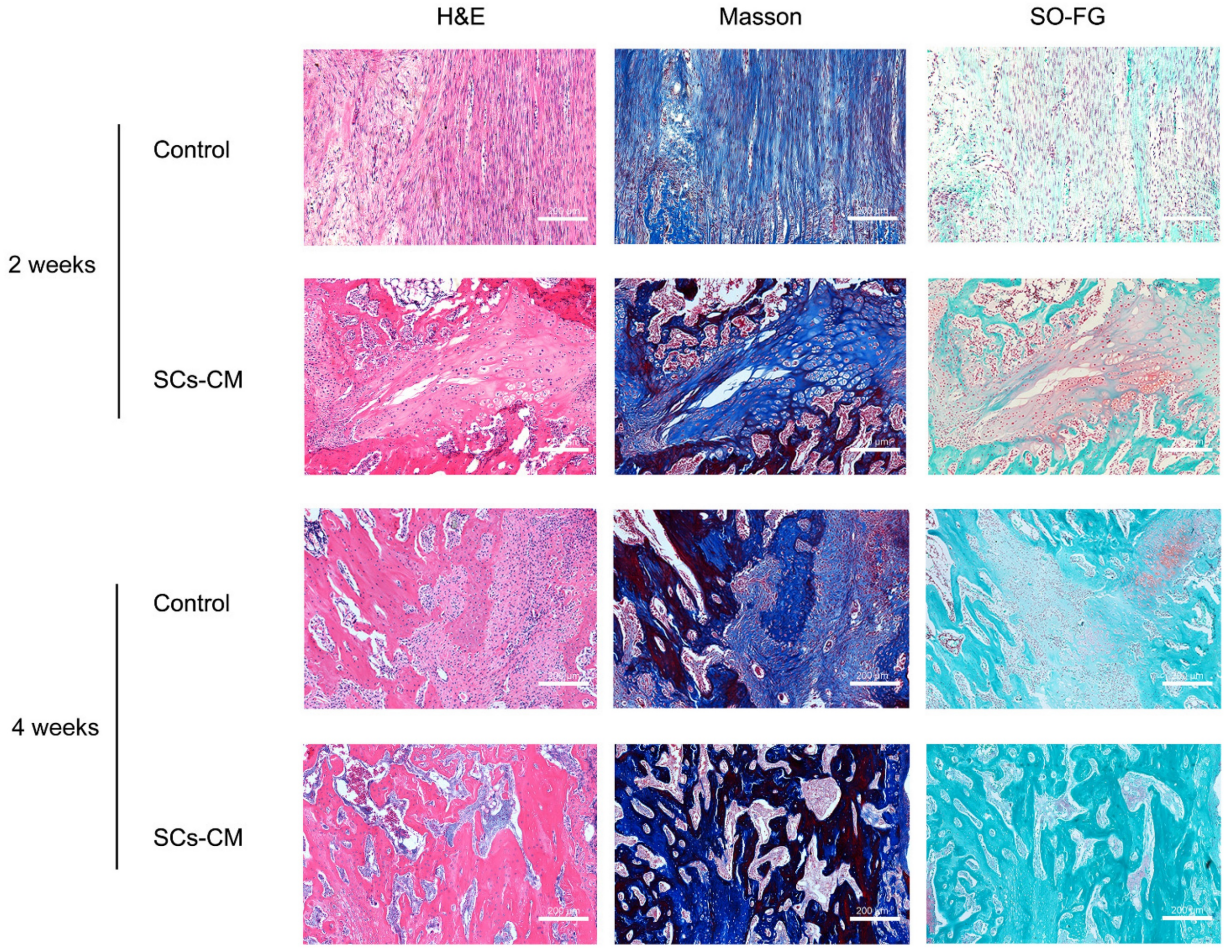
BMSCs-RSC-96 derived CM facilitates vascularized bone consolidation at distraction zone. Distraction regenerate obtained at the 2^nd^ and 4^th^ week of the consolidation phase in the control and SCs-CM groups underwent SOFG, Masson's trichrome, and H&E staining. Scale bar: 200 µm.

**Figure 8:**
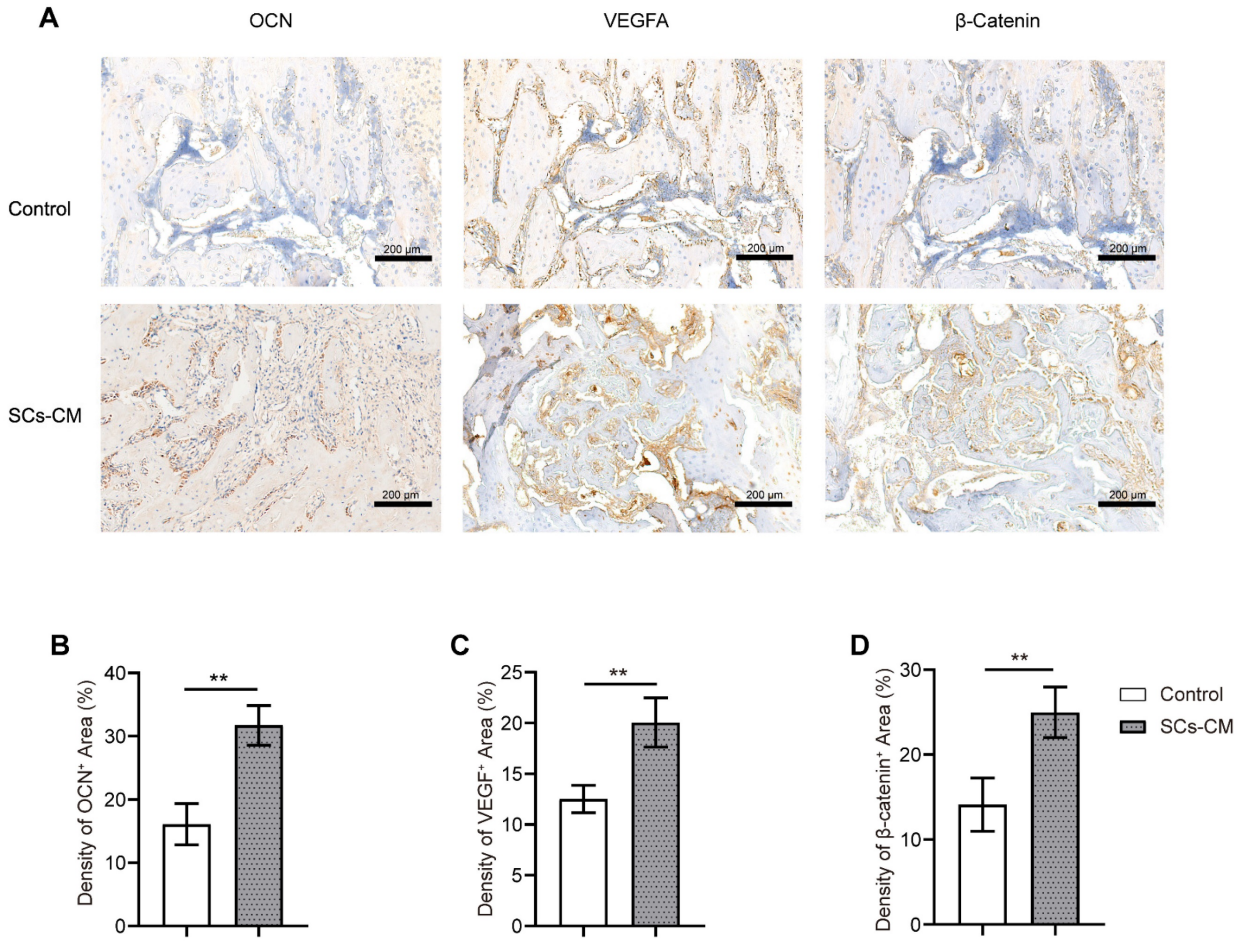
BMSCs-RSC-96 derived CM facilitates vascularized bone consolidation at the distraction zone. **(A)** Immunohistochemical staining of OCN, VEGF, and β-catenin expression in the distraction regenerates of the control and SCs-CM groups. Scale bar: 200 µm. **(B-D)** Local protein expression of OCN **(B)**, VEGF **(C)**, and β-catenin **(D)** via quantitative analysis of the immunohistochemistry area. The results for the control and SCs-CM groups were provided as mean ± standard deviations and were evaluated by Mann-Whitney U-test. *P < 0.05; **P < 0.01.

At the distraction area, various amounts of freshly-developed fibrous-like tissue, cartilaginous tissue, and trabecular bone, were observed after Masson's trichrome, H&E, and SO-FG staining (Figure 7). Newly formed tissue was parallel with the distraction stretch. After 2 and 4 weeks of consolidation, distraction regenerates treated by BMSCs-RSC-96 derived CM exhibited enhanced bone mineralization compared to control group, as revealed by less cartilaginous and fibrous-like tissue, more trabecular bone (Figure 7) and higher OCN expression (Figure 8A, B) in the SCs-CM group. Notably, after 4 weeks of consolidation, an increased intensity of OCN staining within the distraction regenerates, particularly surrounding the newly-developed trabecular bone, was found in SCs-CM group in comparison to control group, indicating the active osteogenesis during the consolidation phase (Figure 8A, B). There was evidently more neovascularization in SCs-CM group as confirmed by elevated VEGF expression in the distraction areas, which verifies the enhanced impact of RSC-96 on the coupled effect of angiogenesis and osteogenesis in BMSCs (Figure 8A, C). Additionally, analysis of immunohistochemical staining showed that the distraction regenerates from SCs-CM group (Figure 8A, D) had more β-catenin expression, which suggests crucial involvement of β-catenin pathway within coupled effect of angiogenesis and osteogenesis induced by BMSCs-RSC-96 derived CM administration during DO.

## Discussion

For the first time, we demonstrated that SCs promoted the pro-angiogenic potential and osteogenesis of BMSCs *in vitro*, and the strengthened effect was attributed, at least partially, to the activated AKT/GSK-3β/β-catenin signaling. Additionally, bone regeneration and neovascularization were promoted via administration of BMSCs-RSC-96 derived CM in a rat DO model.

Neurophysiological regulation is closely intertwined with bone metabolism. The microstructure of normal bone tissue includes basic multicellular units, Haversian canals, and interwoven networks of nerve fibers and blood vessels 30. Sensory and autonomic nerves in bone tissue not only transmit signals of skeletal pain and regulate bone remodeling, but also indirectly participate in regulating skeletal metabolism through blood flow control and secretion of nerve growth factors and neurotrophic proteins 15. Previous studies mainly studied the involvement of SCs in nerve repair 16, 22, whereas no research studied the effect of SCs on pro-angiogenic and osteogenic potential of BMSCs.

In the coculture system of our study, we found that BMSCs proliferation was promoted until the quantity of RSC-96 reached a certain level, suggesting that a certain amount of SCs can coexist well with BMSCs in the local osteogenic microenvironment. Consistent with our results, it has been reported that SCs could facilitate recruitment and extend the lifespan of mesenchymal stem cells (MSCs) for sustained neural regenerative and protective effects 22.

Our further experiments demonstrated at the molecular and genetic levels that RSC-96 could significantly enhance the migration and osteo-mineralization ability of BMSCs in the coculture system. Previous research found that SCs are indispensable for mouse mandibular repair 17 and digital tip regeneration 18. These findings comply with the results of our study. The rich network of blood vessels plays a crucial role in oxygen and nutrients delivery within vascularized bone tissue. Additionally, it regulates a series of biological activities during bone regeneration through the secretion of paracrine factors 4, 31. BMSCs are well-known for pro-angiogenic potential which augments the mineralization of osteoblasts 1, 21, 31. In this study, we incubated HUVECs in each set of CM from BMSCs and found that co-culturing BMSCs with RSC-96 effectively promoted the gene expression related to angiogenesis, with evident formation of capillary networks. We consider BMSCs may serve as a bridge between SCs and vascular endothelial cells, mediating the SCs' role in promoting angiogenesis coupled with osteogenesis.

The paracrine factors of SCs have been widely recognized as the crucial molecules during mouse mandibular repair and digital tip regeneration 17, 18. The nutritional effects of various neurotrophic factors secreted by SCs, such as BDNF, NT-3, and NGF, along with angiogenic factor VEGF, represent as important mediators for regulating the regenerative potential of various precursor cells, including BMSCs. In our study, we found increased secretion level of these neurotrophins in BMSCs-RSC-96 coculture system. Many studies have identified the osteo- and angio-integrative effects of these factors. NT-3 induces VEGF and BMP-2 expression and promotes osteogenesis of MSCs 23, 32. NT-3 can also enhance the proliferation and migration capabilities of HUVECs via the secretion of VEGF, hypoxia-inducible factor 1α, NGF, and BDNF in MSCs, thereby promoting growth plate healing and diabetic wound repair 32, 33. BDNF promotes regeneration of periodontal defects through the trkB (tyrosine receptor kinase B)-cRaf-ERK1/2 signaling pathway 34. Activated ERK signaling has also been reported to mediate the upregulation of VEGF expression and integrin β1-mediated migration of osteoblasts induced by BDNF-trkB for promoting vascularization and fracture healing 24. These findings suggest that the coupling effect of angiogenesis and osteogenesis of BMSCs could be enhanced at least partly by SCs through the paracrine effects of these neurotrophic factors.

The Wnt/β-catenin pathway plays a crucial role in osteogenesis, axon regeneration, and embryonic development 25, 30, 35, 36. Previous research has revealed the significant involvement of the Wnt/β-catenin signaling pathway in SCs proliferation, migration, and myelin sheath formation after peripheral nerve injury 37, 38. Moreover, downregulation of the Wnt/β-catenin signaling pathway can inhibit the proliferation and differentiation of MC3T3-E1 cells after activation of BDNF-related TrkB receptors 28. Exogenous administration of NGF has also been reported to significantly increase load-induced bone formation and Wnt/β-catenin activity in osteocytes 27. These results suggest the potential involvement of β-catenin signaling in the coupled effect of angiogenesis and osteogenesis mediated by SCs.

Numerous studies have noted that AKT signaling is a crucial upstream pathway that mediates the angiogenesis and osteogenesis processes induced by various neurotrophic factors 23, 24, 32. Phosphorylated AKT (Ser473) can activate β-catenin by inactivating and phosphorylating GSK-3β at Ser9, followed by nuclear translocation and transcription of activated β-catenin 26, 39. GSK-3β destabilizes β-catenin by phosphorylating it at Ser33, Ser37, and Thr41 40. Activated β-catenin through phosphorylation at Ser675 and Ser552 has been widely reported to inhibit osteoclastogenesis and promote osteogenesis 21, 25, 26, 30, 35. Phospho(Ser473)-AKT has been reported to be involved in regulating phospho(Ser675)-β-catenin for promoting proliferation of dermal papilla cells, with stimulated secretion of several growth factors 41, 42. A similar mechanism was found in this study. SCs significantly activated the AKT pathway in BMSCs through phosphorylation at Ser473, leading to inactivation of GSK-3β (phosphorylation at Ser9) and activation of β-catenin with increased phosphorylation level at Ser675, thereby enhancing osteogenesis and pro-angiogenic potential. These effects were diminished through AKT inhibitor MK2206. Moreover, MK2206 attenuated the secretions of VEGF, NGF, NT-3 and BDNF in the BMSCs-RSC-96 coculture environment, indicating the AKT-mediated paracrine effect of SCs in the coupled effect of angiogenesis and osteogenesis.

DO involves rhythmic and progressive distraction using external fixation devices following osteotomy 4. The complications led by prolonged wear of external fixation device have always been the great challenge 10, 12. The frequently reported methods for accelerating bone mineralization and reducing the duration of external frame wearing involve local or systemic application of various growth factors 43, 44, stem cell/precursor cell-derived preparations 3, 13, bone regenerative stimulants or other bioactive agents 21, 45. Despite the satisfactory outcomes achieved by cell transplantation, potential biosafety issues such as carcinogenicity, genetic mutations, and thrombus still exist 20. Conditioned medium from seed cell sources can overcome these shortcomings and effectively utilize the paracrine effects. Previous studies have shown that local injection of human BMSC-derived serum-free conditioned medium could accelerate bone consolidation during DO 46, 47. Therefore, we locally applied conditioned medium from SCs and BMSCs coculture system in the rat DO model.

The dynamic expression levels of various molecules during DO provide valuable information for determining the corresponding time point for local administration 3, 45. In our study, local expression of SCs related neurotrophins, including NGF, BDNF and NT-3, gradually increased after osteotomy, peaked at the end of the distraction phase, and decreased afterwards. These findings suggest that traumatic osteotomy and bone lengthening would promote the upregulation of these neurotrophic molecules for tissue regeneration within limited time-period. The latency phase resembles the stage of acute inflammatory response for bone fracture healing, with hematoma formation in the osteotomy area, followed by recruitment of inflammatory cells, and stem cells. During the distraction phase, fibroblasts and collagen fibers align in the direction of tension, with widespread neovascularization for facilitating the initiation of new bone formation 1, 3, 4, 13, 21, 45. Numerous studies have reported that expression levels of various osteogenic and angiogenic markers, including transforming growth factor β1, PDGF, insulin-like growth factor, bone morphogenetic protein (BMP), OCN, and osteopontin (OPN), increased during the distraction phase and decreased upon the beginning of consolidation phase 3, 4, 45. Based on these evidences, this study conducted locally application of the conditioned medium at the beginning of the DO consolidation phase. The additional supplement of conditioned medium ensures the effective level of neurotrophic factors for facilitating the tissue regenerative response. Biomechanical testing, X-ray radiographs and micro-CT data demonstrated that the quality and volume of the regenerates considerably increased after CM administration.

**Figure 9:**
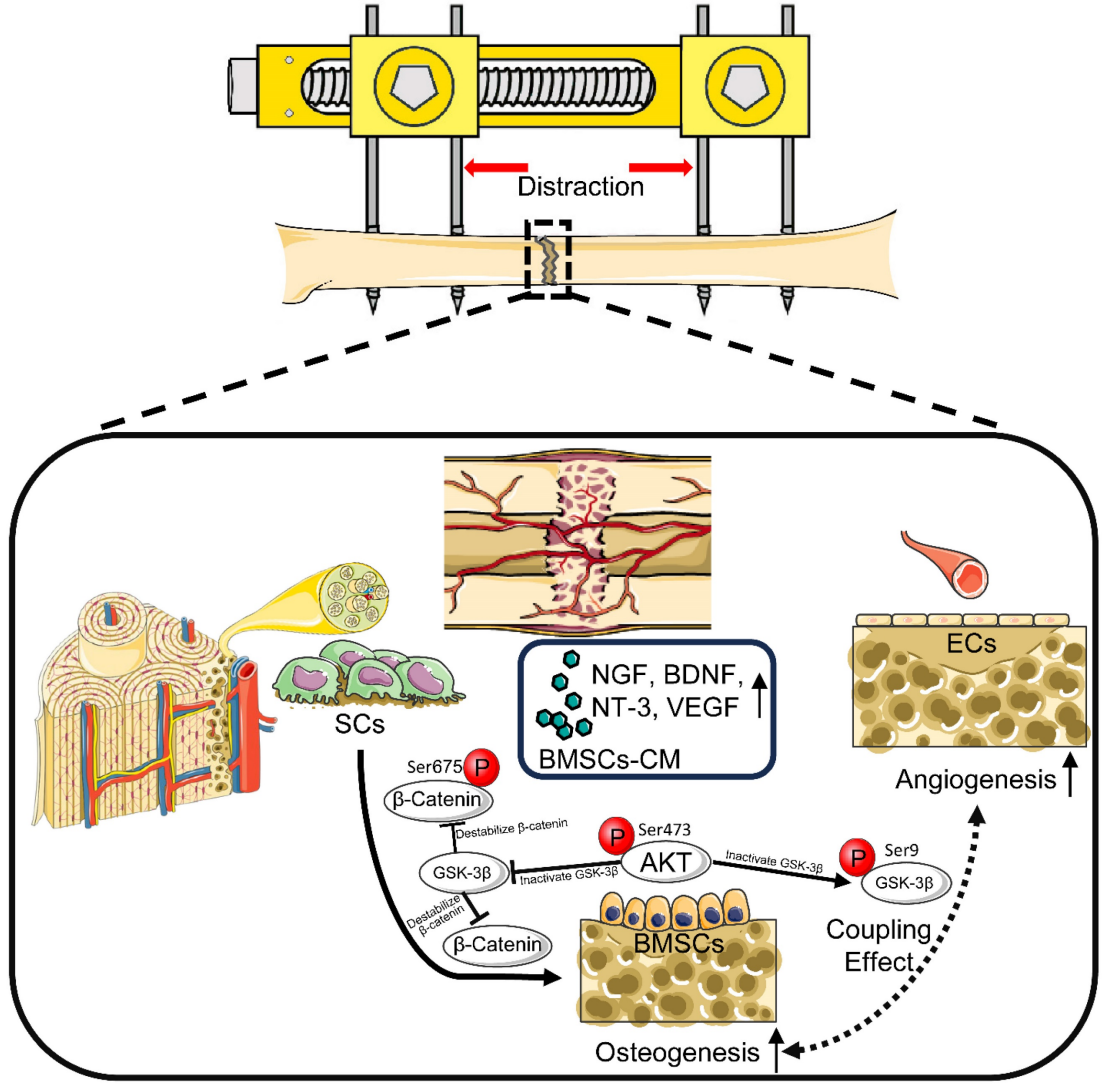
Schematic diagram: SCs induce AKT/GSK-3β/β-catenin signaling for promoting the coupled processes of osteo- and angiogenesis during DO. Under the coculture with RSC-96, AKT/GSK-3β/β-catenin signaling is activated through elevated phosphorylation levels at Ser473 of AKT, Ser9 of GSK-3β, and Ser675 of β-catenin in BMSCs, along with increased production of VEGF, NGF, BDNF, and NT-3. BMSCs' pro-angiogenic potential as well as osteogenic capability are therefore enhanced. In conclusion, through activating AKT/β-catenin pathway and inducing paracrine effect of BMSCs, SCs indirectly improve angiogenesis and directly promote osteogenesis, thereby promoting the coupled process of angiogenesis and osteogenesis for enhancing early bone union in the process of DO.

Histological and immunohistochemical analyses showed accelerated bone formation along with increased local expression of OCN and VEGF, indicating evident signs of osteogenesis and angiogenesis in the SCs-CM group. Furthermore, increased expression of β-catenin in the SCs-CM group validated enhanced bone regeneration by SCs through coupling angiogenesis and osteogenesis via β-catenin signaling. DO enables synchronous elongation and regeneration of various kinds of soft tissue, including muscles, nerves, blood vessels, subcutaneous tissues, and skin 4, 47. Notably, the multi-tissue regeneration potential of SCs involves direct or indirect angiogenic effects 16, 22. The paracrine factors of SCs have been proved to participate in axon regeneration and damaged nerve repair, with increased expression of neurogenic biomarkers such as β-tubulin III, microtubule-associated protein 2, and glial fibrillary acidic protein 16, 48. Based on this, it can be affirmed that SCs may possess a triple effect of neuro-vascular-osseo integration when applied in DO (Figure 9).

Calcium deposition with the regulation of cellular interactions and signaling molecules is crucial for the maturation of robust bone tissue 36. Vascularized and calcified bone callus in the distracted area is considered the critical component for simulating the complex hierarchical structure during the tissue regeneration process of DO 1, 2, 20. Tissue engineering strategies with various cell or bioactive factor delivery scaffolds have been widely used to promote the integration of vascularization and mineralization. Among numerous bone regenerative biomaterials, three-dimensional (3D) bioprinting serves as versatile platforms for fabricating biological scaffolds with multicellular components, spatial biomaterial properties and biomimetic architectures, enabling accelerated healing rate 14, 19, 49. Targeting the nutritional network effects of SCs via 3D bioprinting may facilitate true multilineage regeneration during DO. For clinical translation, future studies should focus on appropriate scaffolds design and explore the intrinsic signaling transmission from SCs to multiple types of precursor cells under the tensile stress of DO.

The present study has several limitations. First, activation of AKT by SCs remains to be fully elucidated both *in vitro* and *in vivo*. Second, a detailed elucidation of the regulatory network between SCs related neurotrophic factors and multiple stem-cell lineages deserves further study for optimized administration during DO. Third, besides AKT/β-catenin pathway, other pathways such as Forkhead box C1 and NFκB may also contribute to enhanced pro-angiogenic and osteogenic effect of BMSCs induced by SCs 21, 37. The detailed mechanism awaits comprehensive exploration.

## Conclusion

SCs mediate the coupling of osteogenic and angiogenic processes in BMSCs via activation of AKT/GSK-3β/β-catenin pathway. In the preclinical rat DO model, application of SCs derived bioactive molecules can effectively promote the coupling of angiogenesis and osteogenesis, which emerges as a promising approach to accelerate tissue regeneration and bone mineralization. In addition, targeting AKT/GSK-3β/β-catenin pathway is a potential treatment strategy during DO therapy.

## Supplementary Material

Supplementary figure.

## Figures and Tables

**Table 1:** The primer sequences used in qRT-PCR.

Gene	Primer sequence forward (5′-3′)	Primer sequence reverse (5′-3′)
ALP (Rattus)	CCGCAGGATGTGAACTACT	GGTACTGACGGAAGAAGG
GAPDH (Rattus)	ATGGCTACAGCAACAGGGT	TTATGGGGTCTGGGATGG
Ang-2 (Rattus)	GAAGAAGGAGATGGTGGA	CGTCTGGTTGAGCAAACTG
OSX (Rattus)	GGAAAAGGAGGCACAAAGAA	CAGGGGAGAGGAGTCCATT
OPN (Rattus)	GGCCGAGGTGATAGCTT	CTCTTCATGCGGGAGGT
Runx2 (Rattus)	ACTTCCTGTGCTCGGTGCT	GACGGTTATGGTCAAGGTGAA
VEGFA (Rattus)	CACGACAGAAGGGGAGCAG AAAG	GGCACACAGGACGGCTTGAAG
BMP-2 (Rattus)	ACTCGAAATTCCCCGTGACC	CCACTTCCACCACGAATCCA
NGF (Rattus)	TGCATAGCGTAATGTCCATGTTG	CTGTGTCAAGGGAATGCTGAA
BDNF (Rattus)	CAGGGGCATAGACAAAAG	CTTCCCCTTTTAATGGTC
NT-3 (Rattus)	CTTATCTCCGTGGCATCCAAGG	TCTGAAGTCAGTGCTCGGACGT

**Table 2:** Antibodies used for Western blot analysis.

Antibody	Product code	Manufacturer	Dilution ratio
AKT	4691	Cell Signaling Technology	1:1000
p-AKT (Ser473)	4060	Cell Signaling Technology	1:2000
GSK-3β	12456	Cell Signaling Technology	1:1000
p-GSK-3β (Ser9)	5558	Cell Signaling Technology	1:1000
β-catenin	8480	Cell Signaling Technology	1:1000
p-β-catenin (Ser675)	4176	Cell Signaling Technology	1:1500
GAPDH	5174	Cell Signaling Technology	1:1500
