# Supplementary figures and images for: Enhanced Bone Regeneration by Schwann Cells through Coupling of Osteogenesis and Angiogenesis via β-catenin signaling in a Preclinical Model of Distraction Osteogenesis
Source: Int J Med Sci. 2025 Jan 1;22(1):209–26. doi: 10.7150/ijms.100854 (PMC11659829; doi:10.7150/ijms.100854)

**Fig. S1. Full-length blots for Fig. 3B**

- 1 OIM
- 2 OIM + RSC-96
- 3 OIM + RSC-96 + MK2206

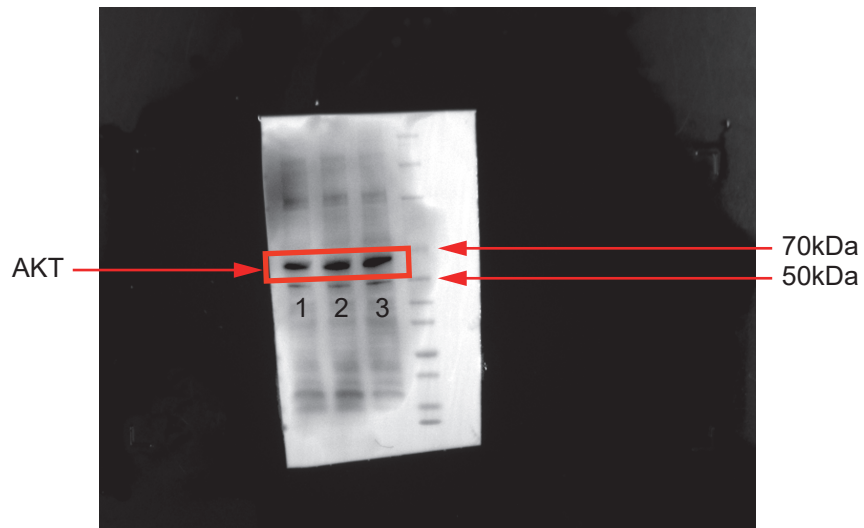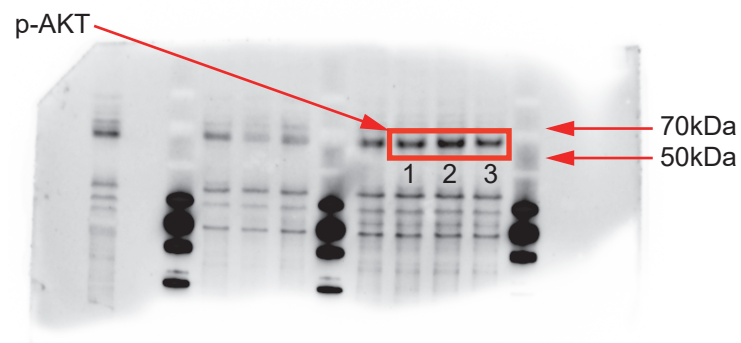

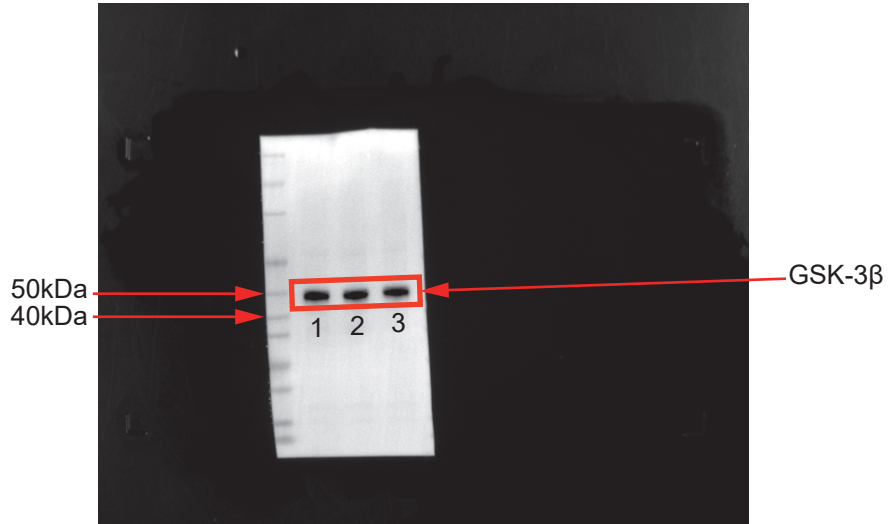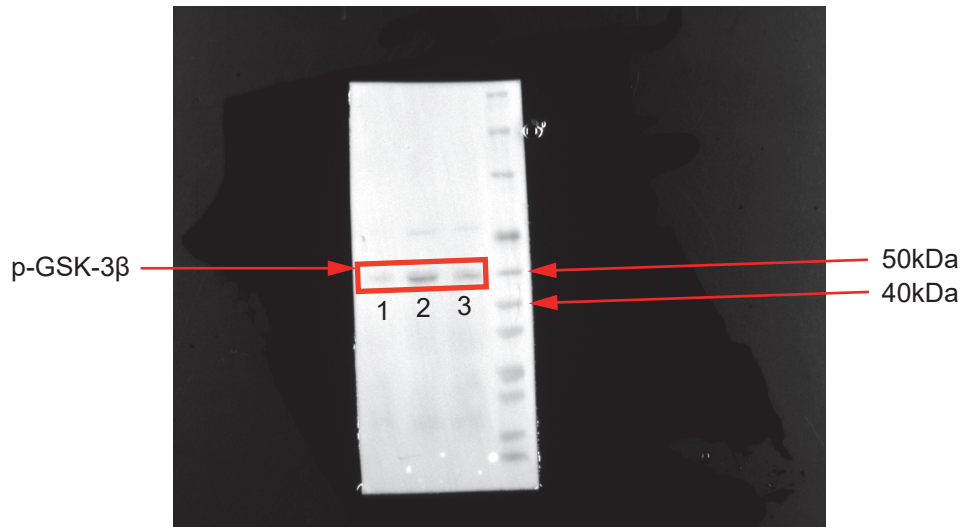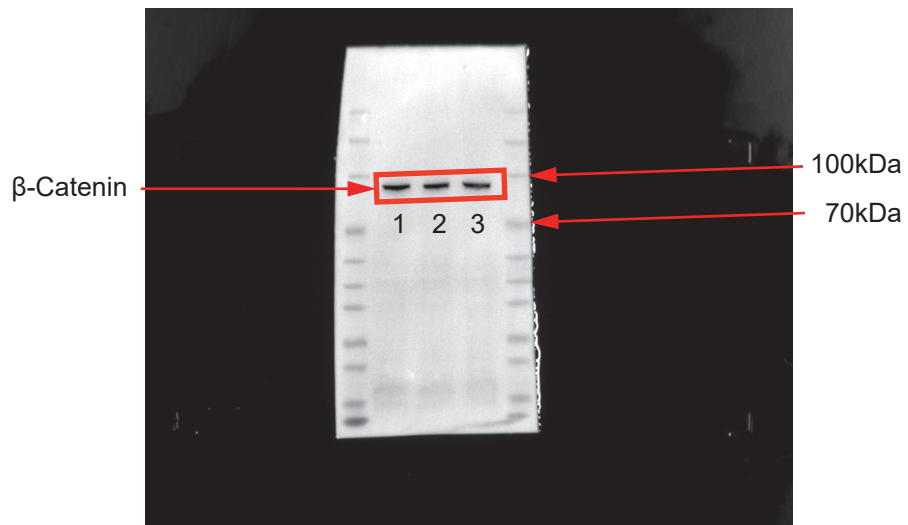

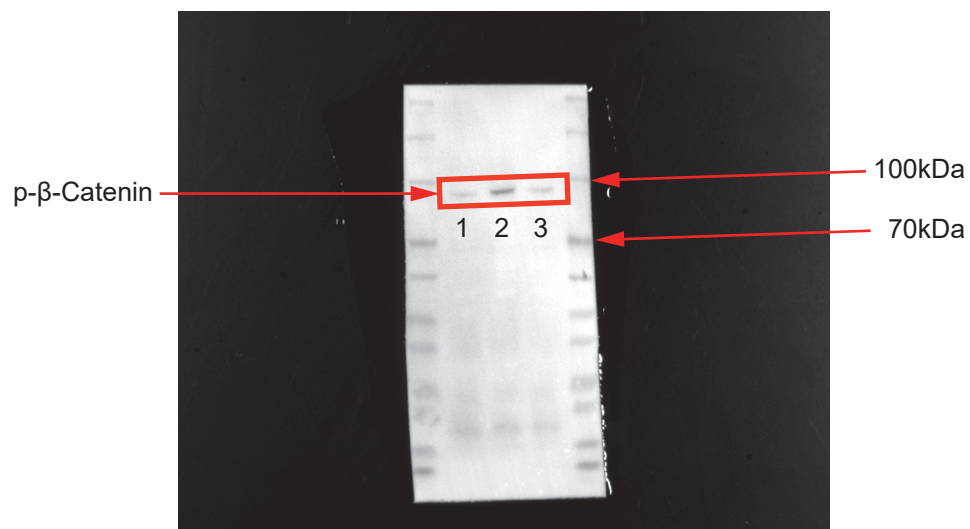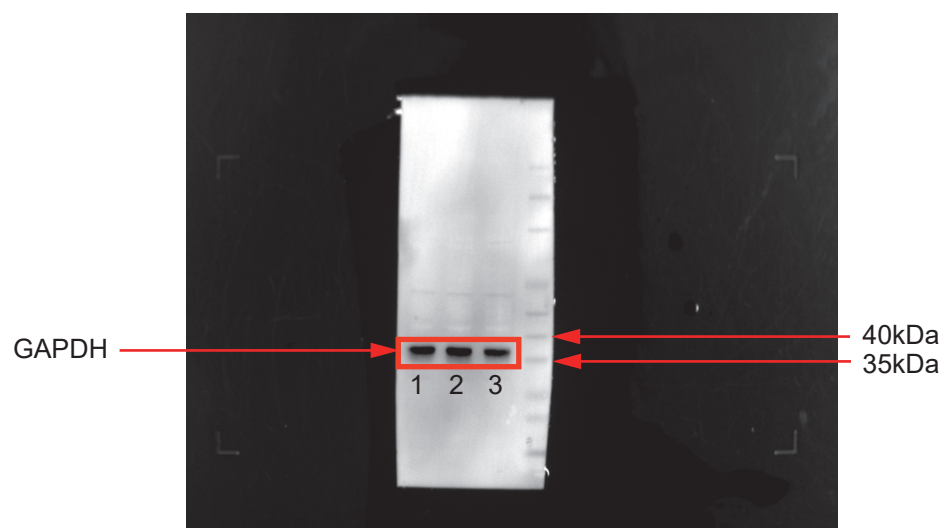

Supplement: Supplementary file 1 — Supplementary figure. [file ijmsv22p0209s1.pdf]
